# State-Dependent Modification of Sensory Sensitivity via Modulation of Backpropagating Action Potentials

**DOI:** 10.1523/ENEURO.0283-18.2018

**Published:** 2018-09-11

**Authors:** Carola Städele, Margaret L. DeMaegd, Wolfgang Stein

**Affiliations:** 1 Institute of Neurobiology, Ulm University, Ulm 89069, Germany; 2 School of Biological Sciences, Illinois State University, Normal, IL 61790

**Keywords:** ectopic action potentials, neuromodulation, sensorimotor, sensory control, state-dependent plasticity, stomatogastric

## Abstract

Neuromodulators play a critical role in sensorimotor processing via various actions, including pre- and postsynaptic signal modulation and direct modulation of signal encoding in peripheral dendrites. Here, we present a new mechanism that allows state-dependent modulation of signal encoding in sensory dendrites by neuromodulatory projection neurons. We studied the impact of antidromic action potentials (APs) on stimulus encoding using the anterior gastric receptor (AGR) neuron in the heavily modulated crustacean stomatogastric ganglion (STG). We found that ectopic AP initiation in AGR’s axon trunk is under direct neuromodulatory control by the inferior ventricular (IV) neurons, a pair of descending projection neurons. IV neuron activation elicited a long-lasting decrease in AGR ectopic activity. This modulation was specific to the site of AP initiation and could be mimicked by focal application of the IV neuron co-transmitter histamine. IV neuron actions were diminished after blocking H_2_ receptors in AGR’s axon trunk, suggesting a direct axonal modulation. This local modulation did not affect the propagation dynamics of en passant APs. However, decreases in ectopic AP frequency prolonged sensory bursts elicited distantly near AGR’s dendrites. This frequency-dependent effect was mediated via the reduction of antidromic APs, and the diminishment of backpropagation into the sensory dendrites. Computational models suggest that invading antidromic APs interact with local ionic conductances, the rate constants of which determine the sign and strength of the frequency-dependent change in sensory sensitivity. Antidromic APs therefore provide descending projection neurons with a means to influence sensory encoding without affecting AP propagation or stimulus transduction.

## Visual Abstract

**Figure:**
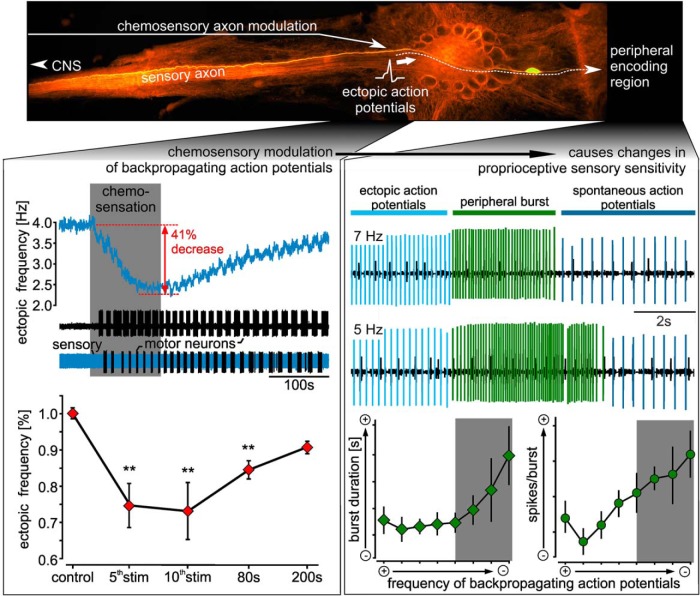

## Significance Statement

Descending modulatory projection neurons are a hallmark of motor systems and fundamentally involved in sensorimotor processing. While they have been shown to interact on many levels with motor networks to dynamically and rapidly adjust motor and behavioral output, their actions on sensory neurons are not well understood. We found that descending projection neurons directly modulate action potential (AP) initiation in a sensory axon, diminishing the frequency of spontaneously generated ectopic APs that propagate antidromically into peripheral sensory dendrites. Changes in the frequency of these backpropagating APs determined the response of the sensory neuron to sensory stimuli. This suggests that descending projection neurons modulate sensory encoding by altering axonal membrane excitability and the frequency of antidromic AP initiation.

## Introduction

While precise and reliable sensory transduction is fundamental for adequate functioning of sensorimotor systems, stimulus properties are not the only factors that contribute to sensory responses. Instead, peripheral and central influences interact to produce the neuronal output, making the state of the system and ongoing activity important contributors to stimulus-induced changes in motor output. A number of mechanisms have been identified that affect sensory responses, including activity- or state-dependent reduction of afferent spike amplitude (Clarac and Cattaert, 1996; Schmitz and Stein, 2000; Margrie et al., 2001; Barrière et al., 2008), spike conduction block (Burrows and Matheson, 1994; Xiong and Chen, 2002; Lee et al., 2012), and regulation of spike initiation (Evans et al., 2003; Cropper et al., 2004). In addition, neuromodulators alter the encoding of sensory information via their actions on local signal processing and transmission, and action potential (AP) initiation (Katz and Frost, 1996; Birmingham, 2001; Birmingham et al., 2003; Mitchell and Johnson, 2003; Dickinson, 2006; Stein, 2009; Nadim and Bucher, 2014). Here, we show that neuromodulators explore an additional pathway to alter sensory encoding, namely via antidromic APs that invade the encoding region of sensory neurons. Neuromodulators can alter axonal membrane excitability and lead to ectopic AP generation, a process common to many systems and neurons, in both normal and pathologic conditions (Dubuc et al., 1988; Léna et al., 1993; Pinault, 1995; Cattaert et al., 1999; Waters et al., 2005; Ma and LaMotte, 2007; Papatheodoropoulos, 2008; Bucher and Goaillard, 2011). These ectopically generated APs functionally add to already present orthodromic APs and correspondingly alter synaptic output (Lambe et al., 2003). Ectopic APs also backpropagate toward the axon origin, carrying potentially important information against the usual propagation direction. This reversal of the functional polarization of the neuron has been implicated to affect encoding of incoming sensory and synaptic stimuli. For instance, backpropagating APs can alter the sensitivity of sensory neurons in crayfish chordotonal organs involved in posture control (Bévengut et al., 1997), in CA1 hippocampal neurons they cause long-lasting synaptic depression of incoming synaptic signals, and they may contribute to memory consolidation (Bukalo et al., 2013).

**Figure 1.**
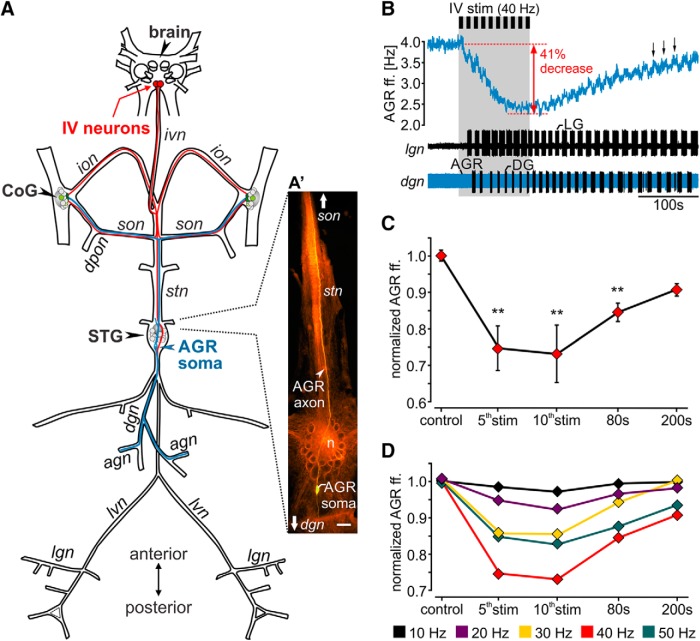
IV neuron activation decreases the frequency of ectopic spike initiation in AGR. ***A***, Schematic of the stomatogastric nervous system. Axonal projections of the paired IV neurons are depicted in red. AGR and its axonal projections are depicted in blue. Nerve names are italicized. Green circles in the CoG represent descending projection neurons. ***A’***, Composite photo of AGR (yellow) and STG (orange) showing the morphology of AGR and its axonal projections in the *stn* and *dgn*. AGR was visualized via intracellular injection of Alexa Fluor 568. Neural structures were visualized via bath-application of the voltage-sensitive dye Di-4 ANNEPS. Note that AGR possesses one to three arbors in the STG neuropil (Städele and Stein, 2016) that are not visible here because of high background fluorescence of the STG neuropil. Scale bar, 100 µm. *n* = neuropil. ***B***, AGR instantaneous ff. (AGR ff., top) and extracellular nerve recordings of the *lgn* and *dgn* (bottom) showing the responses of AGR (blue) and the STG gastric mill neurons before and during IV neuron stimulation (gray area). Black bars above the recording visualize the repetitive stimulation of the IV neurons (40 Hz, 10 consecutive trains). IV neurons stimulation elicited a gastric mill rhythm (note the alternating activity of LG on the *lgn* and DG on the *dgn*) and a concurrent decrease in AGR ff. by 41%. ***C***, Time course of the average change in AGR ff. during 40 Hz IV neuron stimulation for 10 consecutive trains. AGR ff. was normalized to the frequency measured 100 s before IV neuron stimulation (baseline). Control refers to the frequency measured immediately before the stimulation. Shown are means ± SD. *N* = 14 preparations. ***D***, Average time course of the change in normalized AGR ff. during IV neuron stimulation with varying stimulation frequency (10–50 Hz). Shown are means. *N* = 10 preparations. Nerves: *ivn*: inferior ventricular nerve, *ion*: inferior esophageal nerve, *son*: superior esophageal nerve, *dpon*: dorsal posterior esophageal nerve, *stn*: stomatogastric nerve, *dgn*: dorsal gastric nerve; *agn*: anterior gastric nerve, *lvn*: lateral ventricular nerve, *lgn*: lateral gastric nerve. Ganglia: STG: stomatogastric ganglion, CoG: commissural ganglion, brain: supraesophageal ganglion. Neurons: AGR: anterior gastric receptor neuron, IV: inferior ventricular neurons. Panel ***A*** adapted from Hedrich and Stein (2008) and Städele et al. (2012).

While ectopic APs can be initiated by modulators and backpropagating APs can influence stimulus encoding, it remains unclear (1) whether these two processes combined exploit a functional dynamic regulation of stimulus encoding, and (2) what underlying cellular properties facilitate the actions of backpropagating APs on stimulus encoding. We hypothesized that axonal ectopic spiking is directly controlled by neuromodulatory pathways, enabling a dynamic modulation of the processing of incoming stimuli via APs that backpropagate into the stimulus encoding regions of neurons. To test our hypothesis, we used the experimentally advantageous anterior gastric receptor (AGR) neuron in the crustacean stomatogastric ganglion (STG). The STG houses several central pattern generators (CPGs) that are regulated by descending modulatory projection neurons and control aspects of feeding (Stein et al., 2016; Nusbaum et al., 2017; Stein, 2017). AGR senses the tension of the paired gastric mill muscles 1 (*gm1*) when the animal chews food in its stomach. AGR’s soma in the STG protrudes two several centimeter long axons: one to the upstream commissural ganglia (CoG), and one to the peripheral *gm1* muscles (Fig. 1*A*; Combes et al., 1995; Daur et al., 2009). Part of AGR’s activity repertoire is the generation of low-frequency tonic ectopic APs in its central axon that occur as soon as muscle tension is low, i.e., at rest, and in-between bites when bursts are not generated in the periphery (*in vivo* and *in vitro*; Smarandache et al., 2008; Daur et al., 2009).

We show that axonal spike initiation in AGR is directly modulated by descending projection neurons that control the CPGs in the STG. We found that the inferior ventricular (IV) neurons (Christie et al., 2004; Hedrich and Stein, 2008), a pair of descending chemosensory projection neurons that are activated when the animal encounters food as it starts the feeding process (Fig. 1*A*), decrease AGR’s ectopic spike frequency. This effect was mediated via release of histamine, one of the IV neuron co-transmitters, and specific to the ectopic spike initiation zone (SIZ) in AGR’s axon. The change in ectopic spike frequency affected AGR’s sensory burst via antidromic AP propagation into the sensory dendrites, with lower frequencies allowing for stronger sensory bursts. Therefore, an activation of chemosensory pathways primes the proprioceptive system by increasing its sensitivity to muscle tension. We thus demonstrate that frequency regulation of backpropagating ectopic APs by modulatory neurons represents a mechanism to alter sensory encoding.

## Materials and Methods

### Dissection

Adult male crabs (*Cancer borealis*) were purchased from The Fresh Lobster Company and kept in tanks with artificial sea water (salt content ∼1.025 g/cm³, Instant Ocean Sea Salt Mix) at 11°C and a 12/12 h light/dark cycle. Before dissection, animals were anesthetized on ice for 20–40 min. All experiments were performed *in vitro* on isolated nervous systems. The stomatogastric nervous system including the supraesophageal ganglion (brain; Fig. 1*A*) was isolated from the animal following standard procedures, pinned out in a silicone lined (Sylgard 184, Dow Corning) Petri dish and continuously superfused (7–12 ml/min) with physiologic saline (10–12°C). Experiments were performed on fully intact and decentralized preparations. In the latter, the CoGs were removed by transecting the paired *ion* and *son* nerves.

### Solutions

*C. borealis* saline was composed of 440 mM NaCl, 26 mM MgCl_2_, 13 mM CaCl_2_, 11 mM KCl, 11.2 mM Trisma base, and 5 mM maleic acid (Sigma Aldrich); pH 7.4–7.6. In some experiments, low Ca^2+^ saline was used to block chemical transmission in the posterior part of the *stn* (close to the STG neuropil). Low Ca^2+^ saline was composed of 440 mM NaCl, 11 mM KCl, 26 mM MgCl_2_, 0.1 mM CaCl_2_, 11.2 mM Trisma base, 5.1 mM maleic acid, and 12.9 mM MnCl_2_; pH 7.4–7.5. High-divalent (HiDi) was used to raise spike threshold and contained five times the amount of Ca^2+^ and Mg^2+^ than the regular saline. HiDi was composed of 439 mM NaCl, 130 mM MgCl_2_, 64.5 mM CaCl_2_, 11 mM KCl, 12.4 mM Trisma base, and 5 mM maleic acid. HiDi was superfused to the posterior half of the *stn* including the STG neuropil. In these experiments, AGR spike activity was monitored extracellularly on the anterior part of the *stn* (close to the *stn/son* junction) which was not affected by HiDi application. High K^+^ saline was composed of 110–220 mM KCl, 341–231 mM NaCl, 26 mM MgCl_2_, 13 mM CaCl_2_, 11.2 mM Trisma base, and 5 mM Maleic acid; pH 7.4–7.6.

### Modulators and antagonists

Neuromodulators and antagonists were stored as concentrated stock solutions in small quantities at -20°C. Immediately before an experiment, neuromodulators were diluted in saline to the desired concentration. Concentrations varied between neuromodulators and are stated in the text/figures. Histamine dihydrochloride (H7250, Sigma Aldrich) and FMRF-like peptide F1 (TNRNFLRF-NH_2_, Bachem) were diluted in ultrapure water (18.3 MΩ). Cimetidine hydrochloride (PHR1089, Sigma Aldrich) was dissolved in dimethyl sulfoxide (DMSO) and protected from light throughout the length of the experiment. The two orcokinins ([Ala^13^] and [Val^13^] orcokinin; Li et al., 2002) were a gift from Dr. Lingjun Li (University of Wisconsin at Madison, WI).

### Extracellular recordings

If not stated otherwise, all experiments were conducted using non-desheathed nervous system preparations and extracellular recording techniques because removing the sheath of the STG influences modulation of AGR’s ectopic spiking (Goldsmith et al., 2014; Städele and Stein, 2016). For extracellular recordings, petroleum jelly wells were built to electrically isolate a small part of the nerve from the surrounding bath. One of two stainless steel wires was placed inside the well to record neuronal activity of all axons projecting through a particular nerve. The other wire was placed in the bath as reference electrode. Extracellular signals were recorded, filtered and amplified through an AM Systems amplifier (Model 1700). Files were recorded, saved and analyzed using Spike2 Software (CED) at 10 kHz. The activity of AGR was monitored on multiple extracellular recordings simultaneously, namely on the *stn*, *dgn*, and the *son* (Fig. 1*A*). AGR activity was measured as instantaneous firing frequency (ff.) as determined by the reciprocal of the interspike interval.

### Extracellular axon stimulation

We used retrograde extracellular nerve stimulation to activate sensory modalities of interest as described in detail by Städele et al. (2017). In short, a petroleum jelly well was built around a nerve containing the axons of the neurons of interest. One of two stainless steel wires was placed inside the compartment, the other was placed outside. Current pulses were applied with a Master-8 pulse stimulator (A.M.P.I.) controlled by self-programmed Spike2 scripts. IV neurons were activated via extracellular stimulation of the *ivn* with 10 consecutive stimulus trains, 10 to 50 Hz stimulation frequency, 6 s stimulus trains, 6 s intertrain intervals, 1 ms pulse duration, 0.5 to 2 V stimulation voltage (in accordance to their *in vivo* firing pattern; Hedrich et al., 2009, 2011). The ventral cardiac neurons (VCNs) were activated via extracellular stimulation of the paired *dpon* with 10 consecutive stimulus trains, 15 Hz stimulation frequency, 6 s stimulus trains, 4 s intertrain intervals, 1 ms pulse duration, 2 to 3 V stimulation voltage (Beenhakker et al., 2004). In all experiments, both *dpons* were stimulated simultaneously using different channels on the Master-8 stimulator.

To determine history-dependent changes in spike conduction velocity and differences in spike failures before and during IV neuron modulation, AGR was stimulated on the *agn*, a side branch leaving the *dgn* that exclusively contains the AGR axon. To determine changes in spike conduction velocity we used five consecutive trains of 15 Hz stimulation frequency, each train with 28 pulses, 6 to 9 s intertrain interval and 1 ms pulse duration. To elicit spike failures, the *agn* was stimulated with 10 consecutive trains, 10 to 50 Hz stimulation frequency (10 Hz intervals), 9 s stimulus trains, 9 s intertrain intervals, 1 ms pulse duration, and 0.5 to 1 V before and during IV neuron stimulation. We found that the IV neuron-mediated decrease in AGR ff. was strongest after the 5th stimulation train (Fig. 1*C*). We thus started *agn* stimulation after the 5th IV stimulation train to ensure sufficient modulation of the AGR axon. AGR spike conduction velocity was calculated using standard protocols (DeMaegd et al., 2017). To assess the effects of ectopic spike frequency on AGR’s sensory encoding, ectopic APs were elicited via extracellular stimulation of the AGR in the posterior *stn*. A minimum of 20 ectopic APs were elicited and stimulation continued until the first orthodromic spike was detected. Stimulation frequencies were 10 to 3 Hz in 1 Hz steps. Stimulus parameters were 1 ms for pulse duration and 0.2 to 1 V for stimulus amplitudes.

### Drug and saline applications

Neuromodulators, antagonists, HiDi and Ca^2+^ saline were applied selectively to the posterior part of the *stn* where AGR’s ectopic SIZ is located (Städele and Stein, 2016). A petroleum jelly well was used to isolate the application site from the rest of the nervous system. The well had an inner diameter of ∼300–400 µm. Solutions were cooled to 10–12°C and manually applied into the well using a 1 ml syringe with an injection needle. To exclude temperature-induced changes in AGR frequency, saline with the same temperature as the neuromodulators/antagonists was applied 5 min before each application. Measurements for quantitative analysis were taken in steady-state (5 min after neuromodulator/antagonist wash in). Modulators were washed out for 20–40 min with continuous superfusion of cooled saline.

### Sensory burst induction

Either muscle stretch or high K^+^ saline was used to excite AGR’s peripheral dendrites and elicit sensory bursts. In experiments were muscle stretch was used, the *gm1* muscles and their innervation by AGR was left intact. The anterior ossicles where the *gm1* muscles attach to the carapace were pinned in place and the posterior ossicles of the *gm1* muscles were attached to an electrical micromanipulator (PatchStar, Scientifica). Initial muscle stretch was set by adjusting the distance between the posterior and anterior ossicle to the original muscle length measured in the intact animal before dissection. Muscle stretch was applied via a ramp-and-hold movement of the manipulator. Stretch amplitude was adjusted for each animal to activate AGR at physiologically relevant frequencies (typically ∼500 μm). To prevent muscle damage due to many repeats and long duration experiments, muscles stretches were kept short, with a holding phase of ∼500 ms. Stretch rate was ∼200 μm/s.

In experiments where high K^+^ saline was used, an oblong petroleum jelly well with inner diameter of ∼9 × 3 mm was used to isolate the *agn*. Chilled saline was continuously superfused into the well. Inflow and outflow were placed on either side of the *agn*, such that saline flowed in one direction across the *agn*. High K^+^ saline was added to this flow by puffing it into the well for 0.1–0.5 s using a picospitzer. To prevent accumulation of modulator effects puffs occurred every 60–90 s with continuous washouts in between puffs.

Across preparations peripheral bursts triggered by the same concentration of high K^+^ saline were quite variable in burst duration and intraburst ff. Thus, to reduce this variability, the K^+^ concentration was determined separately for each preparation. Specifically, K^+^ saline was applied to the *agn,* and changes in AGR spike frequency were measured on the *dgn*. Puffs started with the lowest concentration (110 mM) and K^+^ concentration was increased until no further increase in intraburst ff. could be observed. After this, the duration of the puff was adjusted so that bursts reflected AGR’s physiologic response to muscle tension. Intraburst ff. was defined as the number of spikes per burst -1, divided by the burst duration.

To determine the influence of stimulation-induced antidromic APs (siAPs) on sensory encoding, the duration of the burst, average intraburst ff., and number of spikes per burst were quantified following different siAP frequencies. siAP frequencies were randomized in each preparation. In each trial, siAP stimulation ended when the first AP of the sensory burst was detected. For each preparation and frequency two trials were averaged in muscle stretch experiments and between one and three trials were averaged in high K^+^ saline experiments. Burst duration was measured as the time from the first orthodromic AP to the last orthodromic AP. Orthodromic APs were identified by spike shape and direction of propagation. Average intraburst ff. was determined as the average of instantaneous ff. during the burst.

### Computer model

To determine the influence that varying ectopic AP frequencies have on sensory bursts, we created a model neuron with standard morphology and passive properties (Ekeberg et al., 1991) using *MadSim* (Stein and Ausborn, 2004; Straub et al., 2004; Städele et al., 2015; freely available for download at http://www.neurobiologie.de). Passive parameters used were: leak conductance, 3 nS, C_m_, 0.8 nF, resting potential, -60 mV. Active membrane properties were implemented according to modified Hodgkin–Huxley equations (Ausborn et al., 2007; Daur et al., 2012). The model can be found on ModelDB (accession number 244260).

A slow K^+^ current (I_KS_) was implemented as non-inactivating current using I = ḡ*α*(V-E). *V* is the membrane potential, *E* the K^+^ equilibrium potential (-80 mV), and *ḡ* the maximum conductance; *ḡ* was set to 0.7 μS. Activation α was calculated using *α = 1/(1+exp((V-*V*_*0*_)/S))*, with *V*_*0*_ = -39.5 mV and *S* = -8 mV. The time constant of activation was constant and varied between models (0.25 to 4 s). Ectopic APs were elicited with 10 ms current pulses (50 nA) at frequencies between 3 and 7 Hz. Sensory bursts were elicited with 12 nA ramp-and-hold current injections (1 s ramp up, 0.5 s holding phase, 2 s ramp down). Simulations produced 20 s long voltage waveforms.

### Experimental design and statistical analysis

All experiments were performed on wild caught male *C. borealis*. Animals were kept for at least 7 days in artificial sea water tanks before experiments. AGR ff. represents the mean instantaneous ff. of APs occurring over 20–40 s. “Baseline” refers to AGR ff. at rest, while “control” refers to AGR’s ff. immediately before a treatment. Data were analyzed using scripts for Spike2 (available from www.neurobiologie.de/spike2) and by using built-in software functions. To determine temporal differences in spike appearance on multiple recording sites, a voltage threshold was used for spike detection and the subsequent maximum or minimum voltage deflection of the signal passing through this threshold was used as trigger. In cases where extracellular stimulus artifacts or the APs of other neurons obscured the neuron of interest, obscuring signals were eliminated from recordings by subtracting the average stimulus artifact with Spike2. Statistical tests were performed using SigmaStat (Systat Software GmbH). Kolmogorov–Smirnov test with Lillifors correction was used to assess normal distribution of data sets. Paired *t* test and one-way repeated measures (RM) ANOVA with Holm–Sidak *post hoc* or Student–Newman–Keuls *post hoc* test were used to test for significant differences. Statistical results are reported as followed: paired *t* test: *t*(degrees of freedom) = *t* value, *p* value, number of preparations; one-way RM ANOVA: *F*(degrees of freedom, residual) = *f* value, *p* value, *post hoc* test, number of experiments (Table 1).

**Table 1.** Statistical tests summary

Figure	Data structure	Type of test	Number of preparations	Output	*p* value
1*C*	Normal	One-way RM ANOVA, Holm–Sidak *post hoc* test	*N* = 14	*F*_(13,4)_ = 49.21	*p* < 0.001 (ANOVA)*p* < 0.05 (*post hoc*)
1*D*	Normal	Paired *t* test	*N* = 10	10 Hz: *t*_(9)_ = 1.72120 Hz: *t*_(9)_ = 3.24130 Hz: *t*_(9)_ = 4.90940 Hz: *t*_(9)_ = 5.36850 Hz: *t*_(9)_ = 3.861	*p* = 0.119*p* = 0.009*p* < 0.001*p* < 0.001*p* < 0.001
3*B*	Normal	One-way RM ANOVA	*N* = 8	*F*_(11,4)_ = 4.17	*p* = 0.06
3*D*	Normal	One-way RM ANOVA, Holm–Sidak *post hoc* test	*N* = 6	*F*_(3,17)_ = 32.36	*p* < 0.001 (ANOVA)*p* < 0.01 (*post hoc*)
4*A*	Normal	Paired *t* test	*N* = 6	*t*_(5)_ = −6.42	*p* = 0.001
4*B*	Normal	Paired *t* test	*N* = 13	*t*_(12)_ = 6.18	*p* = 0.001
4*D*	Normal	Paired *t* test	*N* = 5	*t*_(4)_ = 2.66	*p* = 0.056
4*E*	Normal	One-way RM ANOVA, Holm–Sidak *post hoc* test	*N* = 5	*F*_(4,3)_ = 33.29	*p* < 0.001 (ANOVA)control saline versus intravenous saline: *p* < 0.05 (*post hoc*)control cimet. versus intravenous cimet.: *p* = 0.58
6*C*	Normal	One-way RM ANOVA, Student–Newman–Keuls *post hoc* test	*N* = 8	*F*_(7,42)_ = 3.831	*p* = 0.003 (ANOVA)*p* < 0.05 (*post hoc*)
6*D*	Normal	One-way RM ANOVA, Student–Newman–Keuls *post hoc* test	*N* = 8	*F*_(7,42)_ = 9.717	*p* = 0.003 (ANOVA)*p* < 0.05 (*post hoc*)
6*E*	Normal	One-way RM ANOVA	*N* = 8	*F*_(9,56)_ = 1.198	*p* = 0.315
7*C*	Normal	One-way RM ANOVA, Student–Newman–Keuls *post hoc* test	*N* = 5	*F*_(4,28)_ = 4.65	*p* = 0.001 (ANOVA)*p* < 0.05 (*post hoc*)
7*D*	Normal	One-way RM ANOVA, Student–Newman–Keuls *post hoc* test	*N* = 5	*F*_(4,28)_ = 3.29	*p* = 0.038 (ANOVA)*p* < 0.05 (*post hoc*)
7*E*	Normal	One-way RM ANOVA	*N* = 5	*F*_(4,28)_ = 1.07	*p* = 0.15
9*C*	Normal	Paired *t* test	*N* = 6, *n* = 28 APs	*t*_(27)_ = −0.89	*p* = 0.38
9*E*	Normal	Paired *t* test	*N* = 8	*t*_(7)_ = 0.7	*p* = 0.5

*N* denotes the number of preparations, while *n* is the number of trials/APs. Significant differences are indicated using *p < 0.05, **p < 0.01, ***p < 0.001. Exact *p* values are given unless they were smaller then 0.001, in which case *p* < 0.001 is indicated. *Post hoc* tests were conducted for a significance level of *p* < 0.05 unless otherwise stated. Type of experimental design: Random.

## Results

STG neurons are heavily modulated by descending projection neurons that release a variety of different modulators, including neuropeptides and biogenic amines (summarized in Marder and Bucher, 2001; Selverston et al., 2009; Stein, 2009; Blitz and Nusbaum, 2011). The axons of several neurons are sensitive to biogenic amines in that focal application of these drugs to axonal regions elicits secondary, ectopic SIZs and backpropagating APs (Meyrand et al., 1992; Bucher et al., 2003; Goaillard et al., 2004; Städele and Stein, 2016). Despite this, there is no direct evidence that modulatory control of ectopic APs is indeed employed by projection neurons and that such modulation affects the function of the modulated neuron.

We studied whether the IV neurons, a pair of aminergic descending projection neurons, affect ectopic AP production in the axon of the single-cell muscle tendon organ AGR, and how this modulation affects the encoding of sensory stimuli. AGR and the IV neurons contribute to the same behavior in that they serve complementary functions in feeding. In *C. borealis*, the IV neurons activate rhythmically when the animal encounters food with its antennae (Hedrich and Stein, 2008), usually immediately before food is cleaved by the mandibles and enters the stomach. Activation of the IV neurons causes a long-lasting gastric mill rhythm that drives three internal teeth in mastication within the lumen of the stomach. Once the gastric mill rhythm is running, AGR senses muscle tension of the *gm1* protractor muscles during the powerstroke of the medial tooth and generates bursts of APs near its dendrites in the muscle. Its feedback controls timing and strength of the gastric mill activity (Smarandache et al., 2008). Functionally, thus, IV neuron activity precedes AGR activity, but both can continue throughout feeding. The IV neurons project from the brain through the unilateral inferior ventricular nerve (*ivn*) and innervate the STG and the CoGs (Fig. 1*A*). Importantly, they can be selectively activated by extracellular stimulation of the *ivn* (Hedrich and Stein, 2008), which like chemosensory stimulation of the antennae elicits a gastric mill rhythm.

Part of AGR’s normal activity repertoire is the generation of APs at two different SIZs. Low-frequency (3–9 Hz) ectopic APs are generated tonically in posterior parts of the *stn* (in the trunk of the central AGR axon; Daur et al., 2009; Städele and Stein, 2016). Higher frequency bursts of APs (15–40 Hz) are induced in the peripheral anterior gastric nerve (*agn*), close to *gm1* muscles, and encode muscle tension (Combes et al., 1995; Smarandache et al., 2008). Whenever muscle tension is low, ectopic APs are initiated at the ectopic SIZ in the *stn*, and the peripheral SIZ is silent.

### AGR’s ectopic spike activity is influenced by IV projection neurons

To test whether the IV neurons modulate AGR’s ectopic spiking, we stimulated the *ivn* and monitored AGR’s ectopic spike frequency extracellularly, along with several gastric mill motor neurons. Figure 1*B* shows original recordings of AGR and several gastric mill neurons before and during consecutive *ivn* stimulation with 40 Hz (10 trains, 6 s train/intertrain duration). Stimulation parameters were selected according to the published *in vivo* activity of the IV neurons (Hedrich and Stein, 2008). We observed a strong decrease in AGR’s ectopic ff. in response to IV stimulation, in this particular example from 3.9 to 2.3 Hz (Δf = -1.6 Hz). This diminishment outlasted the stimulation for >300 s.

As previously described (Christie et al., 2004; Hedrich and Stein, 2008), IV neuron stimulation elicited a long-lasting gastric mill rhythm, apparent by the alternating burst activity of the lateral gastric (LG) and the dorsal gastric (DG) neuron (Fig. 1*B*, bottom). Similar to previous observations (Goldsmith et al., 2014; Städele and Stein, 2016), we found that the gastric mill rhythm was accompanied by small rhythmic frequency changes in AGR (Fig. 1*B*, arrows), which are likely to be mediated by the gastric mill motor neurons (GMs) that are active in the same phase as LG. The decrease in AGR ff. could be prolonged when IV neurons were stimulated for longer durations (up to 40 stimulus trains, *N* = 5; data not shown). In these cases, the beginning of the recovery back to baseline frequency was also delayed until the end of the stimulation.

To characterize the IV neuron mediated effect, we measured AGR ff. before IV stimulation (control), during the 5th and 10th stimulation train, and 80 and 200 s after the last stimulation (Fig. 1*C*). On average (*N* = 14), AGR ff. was significantly diminished starting with the 5th stimulation and up to 200 s after the end of IV stimulation, with the largest decrease at the 10^th^ stimulation (from 4.5 ± 1.3 to 3.1 ± 1.1 Hz, Δf = -1.3 ± 0.3 Hz, one-way RM ANOVA, *F*_(13,4)_ = 49.21, *p* < 0.001, Holm–Sidak *post hoc* test with *p* < 0.05). *In vivo*, the response of the IV neurons to chemosensory stimuli at the antennae is quite variable (Hedrich and Stein, 2008), and ranges between 10 and 50 Hz, probably due to the saliency of the chemosensory stimulus. We thus tested a variety of IV neuron stimulus frequencies and found that the response of AGR to IV neuron stimulation depended on stimulation frequency. When we stimulated the IV neurons with frequencies ranging from 10 to 50 Hz, in 10 Hz intervals (Fig. 1*D*), a significant diminishment in AGR ff. was observed at stimulation frequencies of 20 Hz and above (comparison of control and 10th stimulation, paired *t*-test, *p* < 0.009, *N* = 10). Thus, IV neuron ff. determined how strongly AGR ff. was diminished, indicating that the strength of the chemosensory stimulus differentially affects AGR ectopic spiking.

### IV neurons alter AGR’s axonal membrane excitability

**Figure 2.**
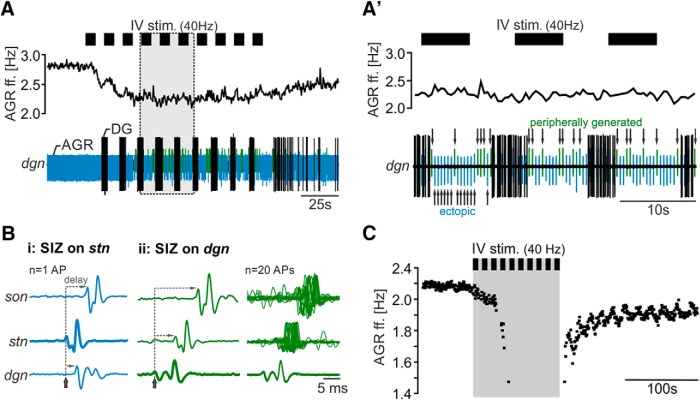
IV neuron stimulation can stop ectopic spike production. ***A***, Extracellular recording of the *dgn* (bottom) and AGR instantaneous ff. (top) showing that AGR spike amplitude changed during strong decrease of AGR instantaneous ff. ***A’***, Magnification of the gray area in ***A***. While AGR spikes on the *dgn* had similar shapes and amplitudes before IV neuron stimulation, spike amplitude continuously changed during IV stimulation. Arrows mark the changes in AGR amplitude. Ectopic APs had negative deflections and are marked blue. APs generated in the periphery had positive deflections and are highlighted in green. Note the different time scales in ***A*** and ***A’***. ***B***, Comparison of spike appearance and delay of arrival of AGR APs with negative (***Bi***), and positive deflection (***Bii***) at three recording sites along the AGR axon (*dgn*, *stn*, and *son*). APs on the *dgn* were used for temporal alignment. Shown are single sweeps (left, middle) and an overlay of 20 APs (right) showing the loss in temporal accuracy (jitter). The recording sites where APs appeared first are highlighted in bold. Colors correspond to different AP deflections as shown in ***A***. Gray lines depict the delay in AP appearance between recording sites. ***C***, Example recording showing a complete stop in AGR’s ectopic firing during IV stimulation (gray area). Note the large gap in spike frequency after the 4th IV stimulus train.

Only one of the two AGR SIZs is active at a given time. The current hypothesis is that the ectopic SIZ has the higher intrinsic membrane excitability and thus dominates AGR’s firing in the absence of sensory spikes (Daur et al., 2009). However, when the excitability at the ectopic SIZ is artificially reduced, the location of spike initiation switches, and the sensory SIZ in the *agn* starts spiking even in the absence of muscle tension (Daur et al., 2009; Städele and Stein, 2016). In some of our experiments, we observed a switch in spike initiation from the *stn* to the periphery during IV stimulation. In nine of 45 experiments, the decrease in AGR ectopic ff. was so dramatic that spike initiation switched from the *stn* to the *dgn*. Figure 2*A* shows the switch in AGR spike initiation exemplarily for one preparation. In this particular example, IV neuron activation initially diminished AGR ff. by ∼25%, until a steady state was reached. At that time, spike polarization of AGR on the *dgn* changed noticeably (Fig. 2*A*’, arrows), indicating that the direction of AP propagation switched and APs were initiated elsewhere (described by Daur et al., 2009; Städele and Stein, 2016). To determine where APs were initiated, we compared the delay in spike appearance between multiple extracellular recording sites along the AGR axon (peripheral: dorsal gastric nerve (*dgn*), central: stomatogastric nerve (*stn*), close to the CoGs: superior esophageal nerve (*son*)). We found that spikes with negative deflection (Fig 2*A*, blue) always occurred first on the *stn* recording site (Fig. 2*Bi*), and traveled orthodromically toward the *sons* and antidromically toward the peripheral *dgn*. In contrast, spikes with positive deflection (Fig. 2*A*, green) always occurred first on the *dgn* and propagated only orthodromically toward the *stn* and *sons* (Fig. 2*Bii*). Spike initiation switched back and forth between the ectopic and sensory SIZ, indicating that the membrane excitability of the ectopic SIZs fell below that of the peripheral one. We noticed that during the switches the temporal accuracy of APs was reduced in that APs arrived with slightly different delays (“jitter”) at the extracellular recording sites (Fig. 2*Bii*, right). This may indicate that APs were either not initiated at a fixed location or that propagation velocity varied. In four additional experiments, the diminishing effect of the IV neurons turned off spike initiation completely (Fig. 2*C*). In conclusion, our results so far predict that IV neurons alter spike initiation in the AGR axon most likely by reducing axonal membrane excitability.

### IV neurons control ectopic spiking via release of histamine

**Figure 3.**
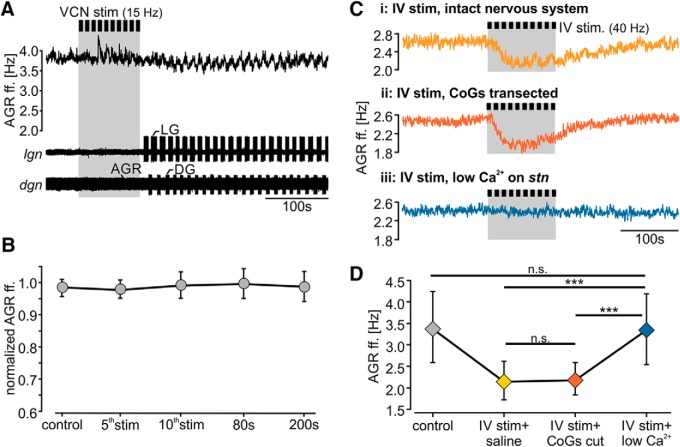
The IV neurons exert their effects on the AGR axon via chemical transmission. ***A***, AGR ff. before and during VCN stimulation (gray bar). VCN stimulation did not diminish AGR ff., but started a gastric mill rhythm (see LG activity on the *lgn* and DG on the *dgn*). Recordings are from the same experiment as shown in Figure 1*B*. ***B***, Average time course of normalized AGR ff. in response to VCN stimulation. Shown are mean ± SD. VCN stimulation did not cause a significant change in AGR ff. *N* = 12 preparations. ***C***, AGR ff. during IV neuron stimulation in the intact nervous system (***Ci***), after CoG transection (***Cii***), and after block of chemical transmission via application of low Ca^2+^ saline to the posterior *stn* (***Ciii***). Recordings are from the same preparation. ***D***, Analysis of the average change in AGR ff. during IV neuron stimulation in saline (IV stim), after CoG transection (IV stim + CoG cut), and after chemical transmission was blocked (IV stim + low Ca^2+^). Shown are means ± SD. Control refers to the frequency measured immediately before IV stimulation. n.s. = not significant different with *p* > 0.8, one-way RM ANOVA, *N* = 6 preparations.

The IV neurons exert their actions on the STG motor circuits directly via histaminergic inhibition of STG circuits, and indirectly via activation of other modulatory projection neurons in the CoG (Christie et al., 2004; Hedrich et al., 2009). Their latter actions are required for eliciting the gastric mill rhythm and involve at least two identified CoG projection neurons (Christie et al., 2004; Hedrich et al., 2009, 2011). Since the observed decrease in AGR ff. during IV neuron stimulation was always accompanied by a gastric mill rhythm, we wanted to exclude that the decrease was due to the involvement of CoG projection neurons. To test this, we elicited gastric mill rhythms that involved the same two identified projection neurons known to mediate the IV neuron-induced gastric mill rhythm. In this case, however, the rhythm was activated via stimulation of the mechanosensory VCNs (Beenhakker et al., 2004). While the elicited gastric mill rhythm (Fig. 3*A*) was accompanied by small rhythmic ff. changes in AGR during the activity phase of the GM neurons (Goldsmith et al., 2014; Städele and Stein, 2016), VCN activation did not cause a significant decrease in AGR ff. This was true for all preparations tested (Fig. 3*B*; one-way RM ANOVA, *F*_(11,4)_ = 4.17, *p* = 0.06, *N* = 8). With the exception of the small rhythmic ff. changes, AGR ff. remained unaffected during and after VCN stimulation. This indicated that the decrease in AGR ff. was specific to the IV neurons and not dependent on the activation of CoG projection neurons.

To further scrutinize this result we completely removed both CoGs by transecting the *ions* and *sons*. This kept the direct connection between IV neurons and STG intact but eliminated indirect effects via CoG neurons. Figure 3*C* shows the decrease in AGR ectopic ff. in response to IV neuron stimulation before and after CoG transection. Across preparations (Fig. 3*D*) the decrease in AGR ff. did not significantly change when the CoGs were transected (comparison of AGR ff. after 5th stim, one-way RM ANOVA, *F*_(3,17)_ = 32.36, *p* < 0.001, Holm–Sidak *post hoc* test with *p* < 0.01, *N* = 6). However, as expected, without CoGs IV neuron stimulation did not elicit a gastric mill rhythm, and the small gastric mill-timed AGR rhythmic frequency changes were absent.

**Figure 4.**
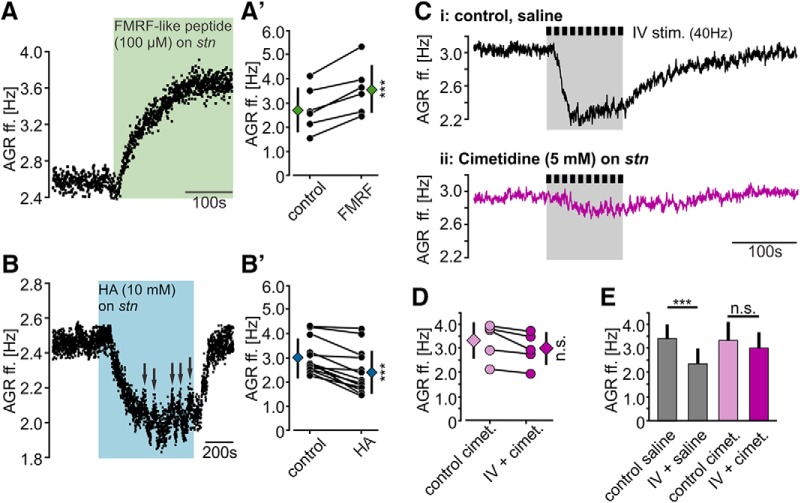
The IV neuron co-transmitter histamine diminishes the AGR ff. mainly via acting on H*_2_* receptors. ***A***, ***B***, AGR ff. in response to (***A***) FMRF-like peptide F1 and (***B***) histamine application to the posterior *stn*. Colored areas mark the time of drug application. Arrows in ***B*** indicate switches of AP initiation to other locations. ***A’***, ***B’***, Analysis of the change in AGR ff. before and during application of (***A’***) FMRF-like peptide F1 and (***B’***) histamine. Black circles represent individual experiments. Diamonds represent mean ± SD. *N* = 6 (FMRF-like peptide F1), *N* = 13 (HA). ***C***, AGR ff. in response to 40 Hz IV neuron stimulation in saline (***Ci***) and after blocking of H_2_ receptors with cimetidine (***Cii***). Recordings are from the same preparation and scaled identically. ***D***, Comparison of AGR ff. during H_2_ receptor blockade with cimetidine immediately before (control cimet.) and during IV stimulation (IV + cimet.). Circles represent individual experiments. Diamonds represent mean ± SD. n.s. = not significant different, paired *t* test, *t*_(4)_ = 2.66; *p* = 0.056, *N* = 5 preparations. ***E***, Average change in AGR ff. in saline (gray) and cimetidine (purple). Shown are means ± SD. One-way RM ANOVA, *F*_(4,3)_ = 33.27, *p* < 0.001, Holm–Sidak *post hoc* test with *p* < 0.05, *N* = 5 preparations. n.s. = not significant different with *p* = 0.58.

To test whether the decrease in AGR ff. during IV neuron stimulation could be chemically transmitted, we blocked chemical transmission at AGR’s ectopic SIZ by reducing the extracellular Ca^2+^ concentration. Specifically, we focally applied low Ca^2+^ saline to the posterior part of the *stn* (close to the STG neuropil edge). Low Ca^2+^ saline prevented the IV neuron-induced decrease in AGR ff. in all experiments (*N* = 6; Fig. 3*Ciii*, *D*), suggesting that the IV neuron effect is mediated chemically. Besides histamine, the IV neurons contain the co-transmitter FMRF-like peptide F1 (Christie et al., 2004). Furthermore, Li et al. (2002) suggested that the IV neurons might also contain different orcokinin isoforms. To test whether any of the identified IV co-transmitters can mediate the observed diminishment in AGR ff., we bath applied them individually at different concentrations to the posterior part of the *stn,* where AGR’s ectopic SIZ is located. Figure 4*A*, *B* show AGR’s response to modulator application. Measurements were taken in steady state, i.e., 5 min after application. Application of 100 µM FMRF-like peptide F1 to the posterior part of the *stn* excited AGR and elicited a strong increase in ff. (Fig. 4*A*). This increase was concentration dependent: 1 µM FMRF-like peptide already caused an increase in AGR ff., but the effect was small (data not shown). On average we found that 100 µM FMRF-like peptide caused a significant increase in AGR ff. by 32 ± 15% (paired *t* test, *t*_(5)_ = -6.42, *p* = 0.001, *N* = 6; Fig. 4*A’*). In all cases, the time constant of the increase was slow and steady state was reached after ∼120 s. Application of histamine (10 mM), in contrast, caused a strong diminishment in AGR ff. (Fig. 4*B*) with a similar time course as seen during IV neuron activation. On average, histamine caused a significant decrease in AGR ff. by 28 ± 9% (paired *t* test, *t*_(12)_ = 6.18, *p* = 0.001, *N* = 13; Fig. 4*B’*) that outlasted the application by ∼250 s. In five out of 13 experiments, the decrease in AGR ff. was strong enough to switch the site of ectopic spike initiation to the *dgn* (Fig. 4*B*, arrows). In none of our experiments (*N* = 5) did application of any of the two orcokinin isoforms tested ([Ala^13^] and [Val^13^], 1–100 µM; Li et al., 2002) elicit a change in AGR ff., despite the fact that both isoforms influenced the pyloric and gastric mill rhythms when applied to the STG (data not shown).

To validate that release of histamine from the IV neurons caused the diminishment of AGR’s ff., we used cimetidine to block histaminergic actions during IV neuron stimulation. Cimetidine is an H_2_ receptor antagonist known to diminish IV neuron-mediated histaminergic effects in the STG (Christie et al., 2004). Specifically, we stimulated the IV neurons in regular saline, observed the decrease in AGR ff., applied cimetidine (5 mM) to AGR’s ectopic SIZ in the posterior *stn,* and then stimulated the IV neurons again. Figure 4*C* shows the response of AGR to IV neuron stimulation before and during cimetidine application. AGR’s decrease was strongly reduced in cimetidine (trace ii), indicating that histamine release contributed to the IV neurons’ effect on AGR. Across preparations (Fig. 4*D*, *E*), we found that in the presence of cimetidine IV neuron stimulation no longer diminished AGR’s ff. (paired *t* test, *t*_(4)_ = 2.66, *p* = 0.056, *N* = 5). In summary, thus, our results demonstrate that histamine released from the IV neurons diminished AGR ff., likely via H_2_ receptor activation.

### IV neuron modulation of the AGR axon is spatially restricted

**Figure 5.**
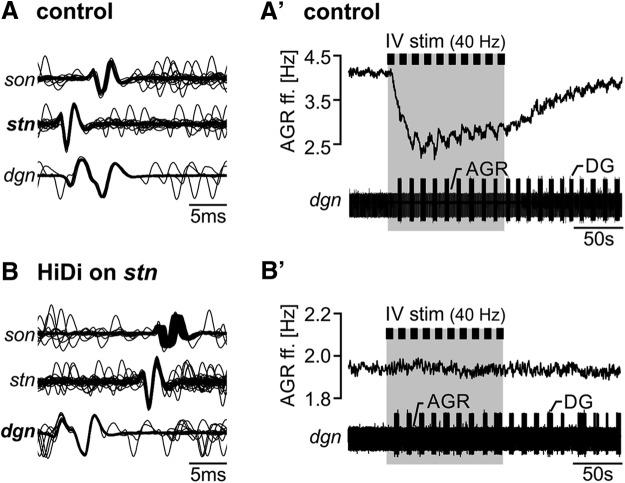
IV neurons only influence AGR when APs are generated ectopically in the *stn*. ***A***, ***B***, Overlay of multiple AGR APs (72 APs each) for (***A***) control condition and (***B***) during HiDi application. Data are from the same preparation. Bold highlighted nerve names mark the recording site where APs appeared first. APs on the *dgn* were used for temporal alignment. ***A’***, ***B’***, Example recording showing AGR ff. before and during IV neuron stimulation for (***A’***) control condition and (***B’***) during HiDi application to the *stn*. Extracellular recordings of the *dgn* (bottom) show the gastric mill rhythm (rhythmic firing of DG). Data from the same preparation as shown in ***A***, ***B***.

If the IV neuron-elicited decrease in AGR ff. is specific to ectopic APs and their initiation site in the axon, it should not affect APs generated in the sensory dendrites. To test this, we artificially moved the site of spike initiation away from the posterior part of the *stn* to the *dgn* via focal application of high-divalent saline (HiDi) to the *stn*. HiDi diminishes membrane excitability, thereby inactivating AGR’s ectopic SIZ and allowing the peripheral SIZ to become active (Daur et al., 2009; Städele and Stein, 2016). Figure 5*A*, *B* show an example of this switch in SIZs. We then activated the IV neurons to test whether AGR ff. is still diminished when APs are initiated in the peripheral dendrites. Similar to the control experiments in regular saline, IV neuron stimulation in HiDi elicited a strong and long-lasting gastric mill rhythm, indicating that the stimulation was successful. Yet, with the peripheral SIZ active, AGR ff. did not decrease anymore (compare Fig. 5*A’* and 5*B’*). We found this to be true for all preparations tested (average change in AGR ff. in comparison to baseline: Δf control = 1.68 ± 0.58 Hz, Δf HiDi = 0.02 ± 0.06 Hz, *N* = 5). We also observed that the small gastric mill-timed oscillations of the AGR ff. were absent when APs where initiated in the peripheral dendrites. In conclusion, descending IV neuron modulation specifically targeted the ectopic SIZ in the *stn*, allowing a direct regulation of ectopic spike frequency at this site.

### IV neurons modify sensory encoding in AGR

Ectopic APs travel both ortho- and antidromically. While it has been shown that orthodromic APs add to already existing APs and increase a neuron’s spike frequency, the influence of antidromic APs is less clear. Unless collisions with existing APs occur, antidromic APs may propagate into dendritic regions with the potential to affect information encoding (Dubuc et al., 1988; Beloozerova and Rossignol, 2004; Ma and LaMotte, 2007). Synaptic processing in neocortex and hippocampus, for example, is altered by APs initiated at the axon initial segment that backpropagate into dendritic areas and modify subsequent signal encoding (Markram et al., 1997; Wu et al., 2012; Bukalo et al., 2013). Similarly, in chordotonal organs of the crayfish leg, back-propagating APs can change the sensitivity of sensory neurons to leg movements. In our experiments, we have so far found that the chemosensory IV neurons modulate the spontaneous ectopic APs in AGR without fully abolishing ectopic spiking. We thus hypothesized that changes in ectopic frequency (and not just their presence of absence) will alter AGR’s sensory activity. We reasoned that ectopic APs invade the sensory dendrites, where they affect membrane excitability and hence AGR’s sensory burst properties. To test this, we induced sensory bursts in AGR, and then compared the elicited bursts at a range of AGR ectopic spike frequencies. To reduce experimental variability in spontaneous and IV neuron-induced ectopic firing frequencies between animals, we induced ectopic APs via extracellular stimulation of the posterior part of the *stn*. Hereinafter, we use the term siAP to distinguish stimulation-induced ectopic APs from spontaneous APs. We elicited siAPs at frequencies between 3 and 10 Hz, which is the range of ectopic spike frequencies observed *in vivo* (Daur et al., 2009, 2012; DeMaegd and Stein, 2018). To avoid interference of siAPs and spontaneously induced APs we did not decrease the stimulation frequency below 3 Hz.

**Figure 6.**
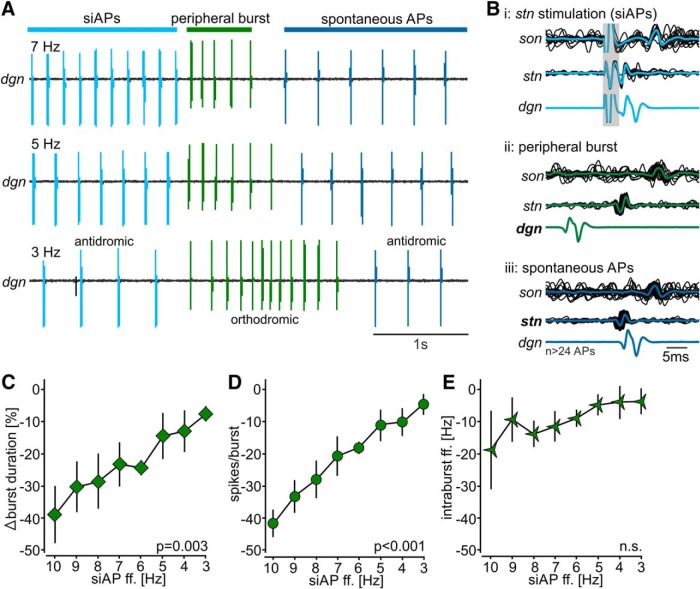
Antidromic ectopic APs alter sensory sensitivity to muscle stretch. ***A***, Original recordings of AGR’s burst activities at different ectopic spike frequencies (7, 5, 3 Hz). Recordings were taken on a section of the *dgn*. Sensory bursts were elicited by stretching the *gm1* muscles. Ectopic APs were elicited with extracellular stimulation of the posterior part of the *stn* (siAPs) and are highlighted in light blue. Orthodromic APs of the sensory burst are depicted in green while spontaneous ectopic APs are highlighted in dark blue. ***B***, Overlay of several original traces from *dgn*, *stn*, and *son* recordings plus average showing the directions of AP propagation for the three conditions shown in ***A***. The gray area depicts the stimulus artifact. ***C–E***, Quantification of sensory bursts at siAP frequencies between 3 and 10 Hz. ***C***, Burst duration. ***D***, Number of spikes per burst. ***E***, Average intraburst ff. Shown are means ± SEM. *N* = 8 preparations each.

We induced sensory bursts in AGR in a semi-intact preparation in which the *gm1* muscles were kept intact, but the rest of the nervous system was dissected as in previous experiments. The muscles were pinned down at the anterior ossicles while the posterior ossicles were attached to a stimulus clamp controlled by an electrical manipulator. We then stretched the muscle to increase muscle tension by pulling the posterior ossicles (see Materials and Methods; Smarandache and Stein, 2007). siAP frequencies were applied in random order to prevent time or hysteresis effects, and each frequency was repeated two times at random intervals. Each siAP frequency trial was preceded by a control muscle stretch with no siAP stimulation, and followed by another control stretch. Muscle stretch was followed by a waiting period of two minutes to minimize potential history-dependent influences of past bursts. This ensured that for each muscle stretch, AGR was in a fully recovered state, i.e., sufficiently rested so that its response was independent of previous stimuli or stretches. As a consequence, however, experiments required the muscles to be healthy for several hours. To minimize muscle fatigue the holding phase of the muscle stretch was kept short (0.5 s). Across animals, the average and maximum frequencies of the elicited bursts ranged from 11 to 25 Hz (average intraburst ff.) and 21 to 37 Hz (maximum ff. in the burst) and were thus within the extent observed in previous *in vivo* recordings of AGR (Smarandache et al., 2008). Figure 6*A* shows an example recording of AGR’s sensory burst on the *stn* following a siAP frequency decrease from 7 to 3 Hz. In this particular preparation, the decrease in siAP frequency caused a prolongation of the sensory burst by 0.78 s (from 0.53 to 1.31 s). As expected, siAPs first occurred on the *stn* and traveled bidirectionally toward the *son* (orthodromic) and the *dgn* (antidromic; Fig. 6*B*i). However, during the sensory burst, APs were elicited in the periphery and only traveled orthodromically from the *dgn* toward the *stn* and *son* (Fig. 6*B*ii). After the end of the sensory burst, AGR regained spontaneous ectopic spike activity. Like the siAPs, these spontaneous APs were initiated on the *stn* and traveled bidirectionally toward the *son* and *dgn* (Fig. 6*B*iii).

Despite our preventative measures, we still observed muscle fatigue and substantial reduction in sensory response over time due to the duration of the experiment. While this could be partially compensated by increasing muscle stretch, it affected baseline sensory responses in AGR. To assess the influence of siAPs on AGR’s response, we thus calculated relative changes (in percentage) elicited by the siAP stimulation with respect to the two control muscle stretches immediately before and after siAP stimulation. Across preparations (*N* = 8), we found that diminishing siAP frequency significantly reduced the change in burst duration from the control burst, signifying prolongation of burst duration with decreasing siAP frequency (one-way RM ANOVA, *F*_(7,42)_ = 3.831, *p* = 0.003, *N* = 8; Fig. 6*C*). *Post hoc* comparison revealed that bursts were significantly prolonged with siAP frequency decreases of 5 Hz or more (Student–Newman–Keuls *post hoc* test, *p* < 0.05, *N* = 8). The number of spikes per burst also followed a similar trend, in which diminishing siAP frequency significantly reduced the difference in the number of spikes per burst from the control burst, signifying an increase in the number of spikes per burst (one-way RM ANOVA, *F*_(7,42)_ = 9.717, *p* < 0.001, *N* = 8; Fig. 6*D*). Spike number decreased continuously from 10 to 3 Hz siAP frequency. *Post hoc* pairwise comparison revealed significant increases in the number of spikes per burst with decreases of siAP frequency of 3 Hz or more (Student–Newman–Keuls *post hoc* test, *p* < 0.05, *N* = 8). By contrast, there was no significant trend for the average intraburst spike frequency (one-way RM ANOVA *F*_(9,56)_ = 1.198, *p* = 0.315, *N* = 8; Fig. 6*E*). This was likely due to burst duration and spike number changing equally.

**Figure 7.**
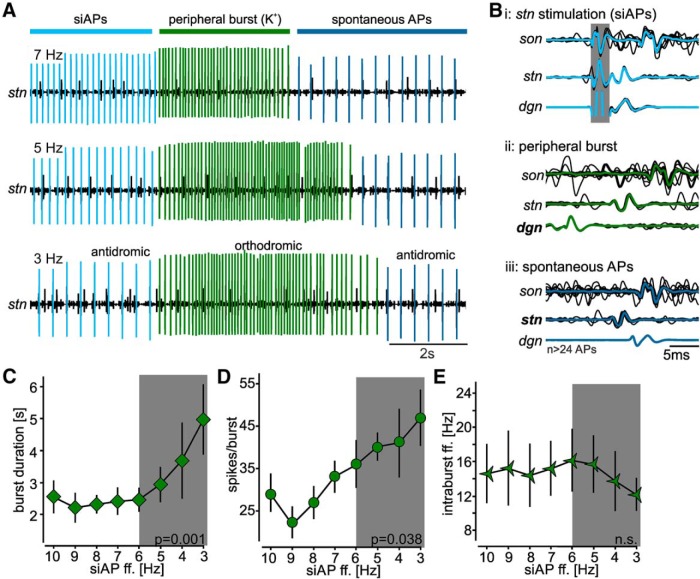
Antidromic ectopic APs alter sensory sensitivity to K^+^ application. ***A***, Original recordings of AGR’s burst activities at different ectopic spike frequencies (7, 5, 3 Hz). Recordings were taken on a section of the *stn,* anterior to the AGR ectopic SIZ. Sensory bursts were elicited with high potassium (K^+^, arrow) in the periphery, ectopic APs with extracellular stimulation of the posterior part of the *stn*. siAPs are highlighted in light blue. Orthodromic APs of the sensory burst are depicted in green, while spontaneous ectopic APs are highlighted in dark blue. ***B***, Overlay of several original traces from *dgn*, *stn*, and *son* recordings plus average showing the directions of AP propagation for the three conditions shown in ***A***. The gray area depicts the stimulus artifact. ***C–E***, Quantification of sensory bursts at siAP frequencies between 3 and 10 Hz. ***C***, Burst duration. ***D***, Number of spikes per burst. ***E***, Average intraburst ff. The gray area indicates the physiologic range of AGR ff. decrease caused by IV stimulation. Shown are means ± SEM. *N* = 5 preparations.

Because we kept muscle stretches short to prevent muscle fatigue, we were unable to account for the effects of ectopic AP frequencies on longer AGR bursts. We thus employed a second approach for initiating sensory bursts. In this case, we focally applied high K^+^ saline to the AGR’s peripheral dendrites. Figure 7*A* shows an example recording of AGR’s sensory burst on the *stn* following a siAP frequency decrease from 7 to 3 Hz. In this particular preparation, the decrease in siAP frequency caused a prolongation of the K^+^ induced sensory burst by 2.9 s (from 3.3 to 6.2 s). Similar to the semi-intact preparation, siAPs traveled bidirectionally toward the *son* (orthodromic) and the *dgn* (antidromic; Fig. 7*B*i), and sensory APs only traveled orthodromically from the *dgn* toward the *stn* and *son* (Fig. 7*B*ii). Spontaneous APs after the sensory burst were again initiated on the *stn* and traveled bidirectionally on the AGR axon (Fig. 7*B*iii). We did not observe substantial reductions in sensory responses over time in these experiments, allowing us to assess AGR responses without further normalization. Across preparations (*N* = 5), we found a significant prolongation of burst duration with decreasing siAP frequency (one-way RM ANOVA, *F*_(4,28)_ = 4.65, *p* = 0.001; Fig. 7*C*). Specifically, K^+^ application elicited the shortest bursts when AGR was pre-activated with 10 to 6 Hz siAP frequency (no significant difference between 10 to 6 Hz). From then on, burst duration steadily increased with smaller siAP frequencies until the siAP frequency reached 3 Hz. Burst prolongation was significant for decreases of 2 Hz or more (Δduration from 3 to 7 Hz = 2.6 ± 2.3 s, 6 to 3 Hz = 2.5 ± 2.2 s, 5 to 3 Hz = 2.0 ± 2.1 s; Student–Newman–Keuls *post hoc* test, *p* < 0.05, *N* = 5). The duration of sensory-induced bursts was thus most strongly affected when the ectopic ff. of AGR decreased from 5 Hz (and above) to 3 Hz, which matches the physiologic frequency decrease elicited by IV stimulation.

In addition, the number of spikes per burst increased significantly when siAP frequency was reduced from 7 to 3 Hz (one-way RM ANOVA, *F*_(4,28)_ = 3.29, *p* = 0.038, Student–Newman–Keuls *post hoc* test, *p* < 0.05, *N* = 5; Fig. 7*D*). Similar to the muscle stretch experiments, the average intraburst spike frequency remained unchanged (one-way RM ANOVA, *F*_(4,28)_ = 1.07, *p* = 0.15, *N* = 5; Fig. 7*E*). In conclusion, these data indicate that ectopic APs that penetrate sensory dendrites are capable of altering the duration and spike number of sensory bursts and thus sensory encoding.

### A slow hyperpolarizing current is sufficient to modify sensory bursts

**Figure 8.**
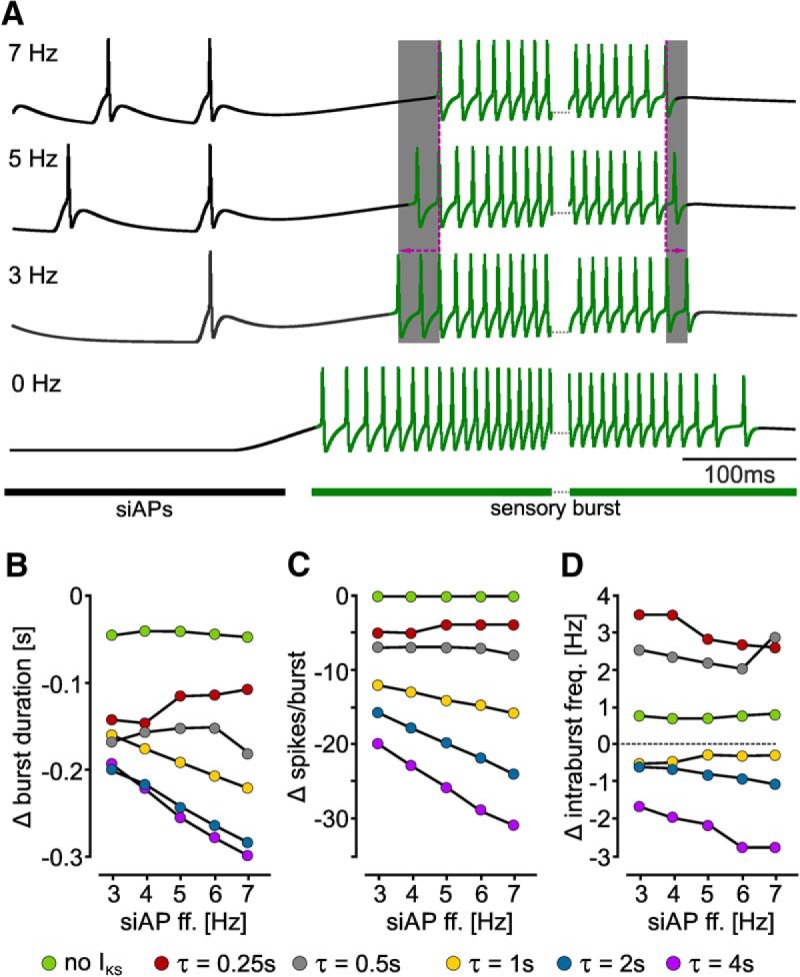
Slow ionic conductances determine the effectiveness of ectopic APs on burst activity. ***A***, Burst activity of three models with an I_KS_ time constant of 2 s. siAP frequency was varied from 7, to 5, to 3 Hz, and compared to 0 Hz (no firing). siAPs are depicted in black, sensory burst APs are highlighted in green. Note that decreasing siAP ff. increased burst duration (gray area). For better visualization, only burst starts and ends are shown. ***B–D***, Analysis of changes in burst structure for different I_KS_ time constants at different siAP frequencies. ***B***, Burst duration. ***C***, Number of spikes per burst. ***D***, Average intraburst ff.

Which properties of the peripheral dendrites allowed ectopic APs to affect the sensory burst? APs that invade stimulus-encoding dendrites can strengthen synaptic input through the accumulation of ions or ionic currents over time (Markram et al., 1997; Wu et al., 2012), or require specific ion channels that affect postsynaptic responses, such as L-type calcium cannels (Bukalo et al., 2013). For neurons that transduce sensory stimuli instead of receiving synaptic input, it is unclear what intrinsic properties might facilitate the modulatory effect backpropagating APs have on sensory responses (Bévengut et al., 1997). Since in AGR the sensory burst was shorter with higher ectopic AP frequencies, we hypothesized that slowly accumulating hyperpolarizing currents may exist in the peripheral dendrites that facilitate this effect. Previous model data had indeed suggested that AGR contains a slow Ca^2+^-activated potassium current (Daur et al., 2012). To address whether a slow hyperpolarizing current is sufficient to enable sensory modulation by ectopic APs, we created a computational model axon using *MadSim* (Daur et al., 2012; Städele et al., 2015). Briefly, we used a single compartment with active properties connected to three passive dendritic compartments to model the peripheral SIZ and the sensory dendrites, respectively. The neuron had a resting potential of -60 mV (similar to AGR; Daur et al., 2012), and thus showed no spontaneous activity. Similar to our physiology experiments, we elicited tonic APs with 7 to 3 Hz (1 Hz step intervals) with pulsed current injections. These stimulation-induced APs represented AGR’s ectopic firing. Sensory bursts were mimicked with ramp-and-hold current stimuli. At least 20 siAPs were elicited before the burst was initiated, and siAPs stopped on burst start. We initially added a slow K^+^ current (I_KS_) with a time constant of activation (τ) of 2 s. We reasoned that to have any effect, the current must accumulate over time, and thus the time constant must be close to the interspike interval of the siAPs. Figure 8*A* shows the change in burst duration of these models for different siAP frequencies. With decreasing siAP frequencies burst onsets occurred prematurely and burst durations were prolonged. This was consistent with our experimental findings in that lower ectopic AP frequencies caused bursts of longer duration.

To test whether I_KS_ was indeed responsible for this observation, we removed it completely from the model, leaving us with a simple Hodgkin and Huxley type neuron (Agüera y Arcas et al., 2003), in which the fast sodium and potassium currents were the only voltage-gated currents. Without I_KS_, bursts in these models were mostly unaffected by the presence of the siAPs (Fig. 8*B–D*), and neither burst duration nor the number of spikes per burst showed a strong dependence on siAP frequency. Thus, these data indicate that a slow potassium current is sufficient to elicit the frequency-dependent modulation of the sensory burst by ectopic APs.

Next, we tested whether having a slow time constant is sufficient to achieve the observed effect. We thus implemented different time constants for I_KS_ activation (τ = 0.25, 0.5, 1, 2, and 4 s). A clear frequency-dependent effect similar to our physiology experiments was only achieved with I_KS_ time constants of 1 s and longer (Fig. 8*B*). With a time constant of 0.25 s, the effect was even opposite to what we had observed in our experiments: decreasing siAP frequencies caused shorter bursts with fewer spikes per bursts (Fig. 8*C*, red trace). In addition, the average intraburst ff. (Fig. 8*D*) was slightly increased, although no frequency-dependent change was obvious. In contrast, with time constants of 1 and 2 s, reducing siAP frequency prolonged the burst, elicited more spikes per burst, and did not alter intraburst firing frequencies. A time constant of 4 s, however, had no further effect on burst duration, but increased the frequency-dependent influence on the number of spikes per burst and caused a slight reduction in intraburst ff.

Taken together, our data suggest that a slowly accumulating K^+^ current is sufficient to elicit the observed changes in the sensory bursts. Furthermore, the strength and frequency dependence of these changes seem to be determined by the time constant of activation, i.e., how fast the I_KS_ accumulated. We used siAP frequencies between 3 and 7 Hz, and thus interspike intervals between 333 and 143 ms. However, only I_KS_ time constants of 1 s and more caused the expected frequency-dependent effect. The model thus predicts that the time constant of the current must be at least several fold larger than the ectopic interspike interval. Modification of stimulus encoding via antidromic APs penetrating peripheral dendrites may therefore represent a general phenomenon in neurons that have slow currents similar to I_KS_.

### IV neurons do not affect AP propagation fidelity along the AGR axon

**Figure 9.**
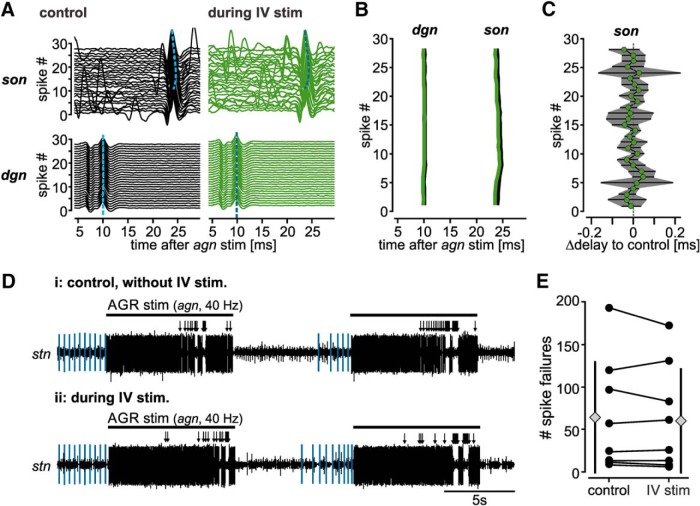
IV neuron modulation does not affect en passant AP propagation. ***A***, Single burst of peripherally initiated AGR APs in response to extracellular stimulation of the *agn* with 28 consecutive pulses and 15 Hz stimulation frequency. Shown are spike appearances on the *dgn* (***Ai***) and *son* (***Aii***) before (black) and during IV neuron stimulation (green). APs were aligned to the stimulus and plotted on top of each other so that the first spike occurs at the bottom. ***B***, Comparison of spike appearance for the example shown in ***A***. Spike times were extracted and plotted as a function of delay to the *agn* stimulation. ***C***, Analysis of the temporal difference in spike appearance on the *son* before and during IV stimulation for 28 consecutive APs. Δdelay is the difference in AP arrival time on the *son* during IV stimulation and the no IV stimulation control. Shown are means ± SD. *N* = 6 preparations, *n* = 28 APs each condition. ***D***, Example extracellular recording of the *stn* showing spike failures (arrows) of AGR APs before and during IV neuron stimulation. Ectopically generated AGR spikes are highlighted in blue while *agn* stimulation induced APs are depicted in black. AGR was activated in the periphery via extracellular stimulation of the *agn*. Recordings are from the same preparation. ***E***, Analysis of the number of spike failures during 10 repetitive *agn* stimulations (40 Hz, 9 s train/intertrain duration, 360 APs/train) before (control) and during IV stimulation. Circles represent data from single experiments; diamonds represent means ± SD, *N* = 8 preparations.

Numerous studies have reported activity-dependent changes in axonal conduction velocities (for review, see Bucher and Goaillard, 2011). In the presence of neuromodulators, propagation dynamics can further change, resulting in modified interspike intervals. This alters AP frequencies as they propagate from the site of spike initiation to the axon terminal, potentially changing transmitter release onto postsynaptic neurons (Lang et al., 2006; Ballo and Bucher, 2009). Since AGR’s sensory APs must travel through the area where the IV neurons diminish axonal membrane excitability, we tested whether the observed decrease in sensory burst duration indeed is faithfully conducted onto postsynaptic neurons during IV-induced axonal neuromodulation. Specifically, we first tested whether axonal conduction velocity of en passant APs was affected by IV activation as they pass the site of modulation. For this, we activated AGR in the periphery and measured the delays at which APs arrived near the CoG, before and during IV stimulation. AGR was stimulated extracellularly on the *agn* with five consecutive trains, each with 28 pulses and 15 Hz stimulation frequency. This approximates AGR’s mean sensory burst frequency *in vivo* (Daur et al., 2009; Daur et al., 2012) and during K^+^ application to the *dgn*. At this frequency, peripheral stimulation overrides all ectopic APs, allowing us to test modulatory effects on the axon without the interference of ectopic APs. Figure 9*A* shows an example of the temporal occurrence of APs on the *dgn* and the *son* in one experiment. APs were aligned to the corresponding stimulus and plotted on top of each other so that the first spike appears at the bottom and the last one at the top. We found that the AGR conduction velocity was history-dependent even in the absence of IV activation in that it initially decreased and then increased (Fig.9*A*, blue lines). For analysis, we plotted resulting AP arrival times as a function of spike number. Figure 9*B* shows that for this particular experiment the pattern of temporal AP appearance during IV neuron stimulation did not change when compared to control condition. This was consistent for all preparations tested (Fig. 9*C*). On average, there was no significant change in AP propagation dynamics during IV stimulation when compared to the no-stimulus control (one-sided paired *t* test, *t*_(27)_ = -0.89, *p* = 0.38, *N* = 6). To conclude, IV neuron modulation of the AGR axon did not influence the temporal dynamics of en passant APs.

However, our experiments were only designed to test for immediate effects IV neurons might exert on AP conduction velocity (∼50 to 100 s after the onset of IV stimulation). Long-lasting neuronal activity has been shown to further modulate axonal excitability and thus AP propagation dynamics (Zhang et al., 2017). The IV neuron effect on these slow mechanisms were not tested in our experiments.

Moderate or high-frequency stimulation (10 to 50 Hz) of axons can lead to propagation failures (Krnjevic and Miledi, 1959; Grossman et al., 1979) so that information is “deleted” along the axon before being conveyed onto postsynaptic partners. Excitability changes in the axon can dramatically affect the rate of conduction failures (Debanne, 2004), and thus change the computational capability of the axon (its “fidelity”). Since the IV neurons diminished the AGR axon excitability, we speculated that the rate of propagation failures and the maximum transmission frequency at which the AGR axon is able to conduct APs is diminished by IV neuron modulation. To test this, we stimulated the *agn* with 10 consecutive trains of 10 to 50 Hz (10 Hz steps) for 9 s and compared the number of spikes passing through the site of modulation before and during IV neuron activation. 50 Hz simulation reliably caused spike failures of >50% (2385 ± 585 pulses out of 4500, *N* = 8) even in the absence of IV neuron activation, indicating that reliable AP propagation in AGR is limited to lower frequencies. Stimulation frequencies between 10 and 30 Hz reliably elicited APs without any spike failures in control and during IV activation. Yet, at 40 Hz spike failures started to occur both in control and during IV neuron stimulation. Figure 9*D* shows an example of AGR’s responses to 40 Hz *agn* stimulation before and during IV neuron activation. However, we found no significant difference between the number of spikes failing during IV neuron modulation and the no-stimulation control (control: 64 ± 68 failures, during IV stim: 62 ± 62 failures, paired *t* test, *t*_(7)_ = 0.7, *p* = 0.5, *N* = 8; Fig. 9*E*). Taken together, our results demonstrate that descending modulation of the AGR axon by IV neurons affected neither maximum transmission frequency nor axonal propagation fidelity.

## Discussion

The sensitivity of many sensory neurons are state- and context-dependent. Consequently, sensory activity does not solely depend on stimulus properties, but also on internal and external conditions of the animal. Backpropagation of APs into the stimulus encoding sites of neurons has been implicated in altering synaptic and sensory stimulus encoding, but to our knowledge, this is the first study to demonstrate that backpropagating ectopic APs dynamically modify sensory encoding in response to modulation by descending projection neurons.

### Neuromodulation of sensory systems

The ability of the neuromodulatory system to globally affect sensory systems has long been established (Birmingham, 2001). The intensity of reflexes such as startle responses, for example, are altered by monoamines, peptides, and opiates (Davis, 1980). Sensory modulation is not limited to reflexes, however, and can affect aspects of social communication (Hoke and Pitts, 2012), taste (Lemon, 2017), olfaction (Kawai et al., 1999), hearing (Brozoski and Bauer, 2016), and pain (Tsuda, 2017). While initially thought to allow the animal to optimize energy expenditure, it is now clear that modulating sensory responses is a common phenomenon that allows organisms to dynamically modify sensory responses in a variety of external and internal conditions.

Contrary to endocrine and paracrine actions, direct and more local control of sensory sensitivity by the CNS is not well understood, with the exception of sensory gating via presynaptic inhibition that leads to a diminishment or even complete block of afferent spike propagation into sensory terminals (El Manira and Clarac, 1994; Coleman et al., 1995; Sauer et al., 1997; Stein and Schmitz, 1999; Beenhakker et al., 2005, 2007; Barrière et al., 2008; DeLong et al., 2009; Bardoni et al., 2013). Our results show that while axonal membrane excitability in a sensory neuron, AGR, was diminished by histaminergic actions of descending projection neurons, afferent spike propagation in AGR remained unaffected (Fig. 9). Specifically, neither AP propagation velocity, history dependence, nor AP failure rate was altered. This occurred despite the fact that global aminergic modulation has been shown to significantly alter AP propagation dynamics in other axons of the same system (Panzeri et al., 2001; Ballo and Bucher, 2009; Ballo et al., 2010, 2012). However, these modulatory influences occur when large stretches of the axon are exposed to modulation (Ballo and Bucher, 2009), which is not the case for modulation by the IV neurons. Instead, modulation appeared to be restricted to the location of the ectopic SIZ itself (Fig. 5), suggesting a different function of modulation besides influencing propagation of sensory information.

Our main result is that descending projection neurons modulate the frequency of backpropagating APs, and that this change in frequency modifies sensory sensitivity. There are many examples in which additional APs are elicited in ectopic locations, i.e., spatially distant from the canonical spike initiation site that supports the main function of the neuron. In STG motor neurons, dopamine can elicit additional APs in the axon trunk (Bucher et al., 2003), in thalamocortical neurons, nicotinic modulation of axon terminals elicits additional APs (Lambe et al., 2003), and clinically therapeutic techniques such as spinal cord stimulation or deep brain stimualtion elicit ectopic APs to alleviate symptoms of neuropathic pain (Li et al., 2006) and Parkinson’s disease (Garcia et al., 2005), respectively. These ectopically generated APs add to already present orthodromic APs and correspondingly alter synaptic output. Similar effects on postsynaptic neurons have been described for sensory neurons as well (Städele and Stein, 2016). However, if ectopic APs are generated at the axon trunk or the axon terminal, they also backpropagate antidromically toward the axon origin, reversing the functional polarization of the neuron by carrying information about distant modulator actions to sites of stimulus encoding where they can affect future encoding events. Previous studies on crayfish chordotonal organs have demonstrated that backpropagating APs indeed can alter the sensitivity of velocity and position-dependent sensory neurons that are involved in leg posture control (Bévengut et al., 1997). The sensory axon terminals are excited by GABAergic primary afferent depolarizations that occur in phase with the CPG that drives leg movements. When suprathreshold, antidromic APs are elicited that have the ability to alter the sensitivity to sensory stimuli (Bévengut et al., 1997). In this case, the range of ectopic spike frequencies, origin of modulation and control of antidromic firing are unclear, as are the mechanisms by which antidromic APs alter sensory sensitivity when they invade the sensory dendrites. Underlying channels have been explored in CA1 hippocampal neurons, where antidromic APs elicited by GABA_A_-mediated depolarization and an additional electrotonic coupling at axon terminals cause long-lasting synaptic depression of incoming synaptic signals, resulting in a rescaling of synaptic weights that may contribute to memory consolidation. Here, L-type calcium channels are a prerequisite for invading APs to have an effect on synaptic integration (Bukalo et al., 2013). While there are no synaptic inputs in sensory dendrites, our models indicated that similarly to the CA1 neurons, passive properties and Hodgkin–Huxley-type sodium and potassium currents are not sufficient to elicit such a response, but a slowly accumulating ionic current was necessary. Specifically, our results show that changes in IV neuron firing rate reduce AGR’s ectopic spiking accordingly, and that this causes a frequency-dependent increase in AGR’s response. A slow hyperpolarizing current was sufficient to elicit this effect in the model. It is reasonable to assume that a slow accumulation of outward currents will hyperpolarize the dendrites, and reduce local excitability to sensory inputs. Interestingly, however, the time constant of the current determined the sign of the response in that a fast time constant reversed the frequency dependence, causing a diminished response with lower ectopic AP frequency. This indicates that the rates of gate opening and closing determine if there are frequency-dependent effects, and if so, which direction they have. The different rates at which similar ion channels operate in different neurons, even within the same organisms, is quite striking. It is conceivable, thus, that those rates are adjusted to serve specific function for given neurons, and in case of backpropagating APs, to allow and regulate effects on stimulus encoding. It is obvious that backpropagating APs may only exert effects if they truly invade the dendritic structures though. This may not always be the case, as antidromic APs in STG motor neurons have little effect on motor pattern generation (Bucher et al., 2003), and neurons may be able to differentiate between orthodromically and antidromically propagating APs (Dugladze et al., 2012).

### Axon modulation and mechanisms

The actions and functions of neuromodulators on axons remain somewhat enigmatic, despite the fact that membranes of myelinated and unmyelinated axon trunks are endowed with ionotropic and metabotropic receptors for transmitters and neuromodulators (summarized by Bucher and Goaillard, 2011; Sasaki et al., 2011). We demonstrate that the IV neurons directly modulate the AGR axon and enable a frequency-dependent change of sensory sensitivity in AGR. Like many other descending projection neurons (Nusbaum, 2013), the IV neurons contain several co-transmitters (Christie et al., 2004; Stein et al., 2007). Two of the IV neuron’s co-transmitters, histamine and FMRF-like peptide, had the ability to alter AGR’s ectopic spike frequency in opposing ways. When applied to AGR’s ectopic SIZ, histamine reduced AGR’s ff. similar as seen during IV neuron activation, while FMRF-like peptide increased the ectopic ff. (Fig. 4). While the AGR axon is responsive to both transmitters, our results indicate that only histamine is neuronally released at AGR’s ectopic SIZ. When histamine actions were blocked, we found no evidence of excitatory actions by the IV neurons on AGR’s ff., indicating that FMRF-like peptide was not released at the *stn*. This is consistent with the observation that IV neuron transmitter actions seem to diverge in that they act on different neuronal structures. It has previously been shown that most, if not all, direct actions of the IV neurons on the STG motor circuits are mediated by histamine, while FMRF-like peptide actions were absent (Christie et al., 2004). On the other hand, IV neuron actions on CoG neurons seem to be exclusively mediated by FMRF-like peptide.

Histamine, just like other biogenic amines, mediates a plethora of different actions on neurons (summarized in Haas et al., 2008). It either acts via metabotropic G protein-coupled receptors (H_1_–H_4_) that mediate an increase in intracellular cAMP, or via ionotropic receptors that activate chloride conductances (Hatton and Yang, 2001; Lee et al., 2004; Cebada and García, 2007). In the stomatogastric nervous system all hitherto described histaminergic actions are direct in that histamine elicits a fast and strong hyperpolarization of pyloric pacemaker neurons in the STG (Claiborne and Selverston, 1984; Pulver et al., 2003; Christie et al., 2004). In our experiments, histaminergic effects on AGR lasted for up to 300 s, indicating that the kinetics are distinct from histaminergic actions on STG motor neurons.

What mechanisms could be responsible for the observed decrease in ectopic spike initiation during IV neuron activity? Potential mechanisms include a reduction in depolarizing ionic conductances or activation of hyperpolarizing conductances (e.g., chloride or potassium) and associated shunting by histamine. We found that blocking H_2_ receptors substantially diminished the IV neuron-mediated effect. H_2_ receptors have been shown to interact with ionic conductance such as I_K(Ca)_ (Haas and Konnerth, 1983; Pedarzani and Storm, 1993) and I_H_ (McCormick and Williamson, 1991; Pedarzani and Storm, 1995). While I_H_ is present in AGR (Daur et al., 2012), I_K(Ca)_ has not been experimentally verified. A histamine-induced decrease of I_H_ or an increase of I_K(Ca)_, or a corresponding shift in their voltage or calcium dependence, could alter cell resting potential, input resistance, and consequently spike activity. For example, Ballo et al. (2010) show that axonal I_H_ depolarizes the resting membrane potential in the axon and causes ectopic spike initiation. However, due to its depolarizing effect, I_H_ also affects AP propagation. In specific, the strength of I_H_ influences the slow after-hyperpolarization (sAHP) of APs during repetitive activity (Grafe et al., 1997; Soleng et al., 2003; Baginskas et al., 2009). The sAHP, in turn, has been implicated in a general slowing of AP propagation (Bostock and Grafe, 1985; Moalem-Taylor et al., 2007; Ballo et al., 2010, 2012). The fact that in our experiments AP propagation during IV neuron modulation did not change (Fig. 9) indicates that if H_2_ receptors modulate I_H_ or another conductance in AGR, this effect must be spatially restricted to the ectopic SIZ.

### Functional implications

The stomatogastric nervous system is an extension of the CNS and controls aspects of feeding (Stein, 2017) that are mediated by striated muscles in the foregut. AGR and IV neuron function are closely intertwined as they both pertain to the gastric mill rhythm, which controls the movement of three internal teeth that masticate food in the stomach. The IV neurons respond to chemosensory stimulation of the antennae, when food touches the mouth or is in close vicinity to it. This is the case immediately before food is swallowed and enters the stomach, and starts the gastric mill rhythm (Böhm et al., 2001; Hedrich and Stein, 2008). In contrast, AGR provides feedback about ongoing gastric mill rhythms, i.e., when food has already entered the stomach and is being chewed. AGR responds to muscle tension in the large *gm1* muscles that carry out the power stroke of the median tooth, and like its mammalian tendon-organ counterparts, reinforces muscle force when resistance to a movement is encountered. Thus, IV neurons and AGR complement each other; IV neurons precede AGR activity at the beginning of feeding, and AGR entraining the rhythm once it is running. Our results show a direct interaction between these two independent sensory pathways. We tested this using semi-intact and isolated nervous system preparation in which the sensory neuron was fully functional, all spike frequencies operated within the previously measured *in vivo* activities, and in which stimulation protocols were exclusively based on measured *in vivo* activity. The IV neurons directly diminish ectopic spiking in AGR, which ultimately leads to an increased sensitivity to muscle tension. AGR’s response is maintained for longer and the number of APs increases. This happens once the IV neurons are activated by food stimuli, before the gastric mill rhythm is started. Chemosensory stimuli thus prime the proprioceptive response of AGR in that its sensitivity to resistance to tooth movements is heightened. Functionally, this may prepare the sensory system in the stomach for incoming food and allow it to adequately and quickly respond to filling of the stomach, and provide the appropriate strength necessary to carry out the chewing movements. Conversely, once all food is ingested, the IV neuron activity is likely to subside, indicating that no further increase in tension may occur, removing the need for AGR to possess heightened sensitivity. Interestingly, the IV neuron response to chemosensory stimuli seem quite variable (Hedrich and Stein, 2008). Our results indicate that this will translate into variable modulation of AGR’s ectopic frequency and that proprioceptive sensitivity will change along with it. The idea that antidromically traveling ectopic APs are under modulatory control by other sensory pathways and alter sensory encoding is a particularly intriguing concept, since it allows these structures to be primed for incoming sensory information detected earlier by other sensory pathways.

Given that descending modulatory projection neurons are a hallmark of most sensorimotor systems (Nusbaum, 2013), neuromodulation of sensory axons may be common to many systems and enable state-dependent changes in sensory encoding or transmission.

The ability of backpropagating APs to alter signal encoding has been demonstrated in the neocortex and hippocampus: APs can backpropagate from the axon initial segment into nearby dendritic areas, resulting in modified signal encoding of subsequent inputs (Markram et al., 1997; Wu et al., 2012). In human C-fibers, APs elicited at the axon initial segment can antidromically invade spatially separated dendrites and reset these dendritic regions. This ensures that only one of the many sensory dendrites dominates the sensory response (called a flip-flop), namely the one with the fastest and highest response to sensory input. While this process seems to be specific to convergence of sensory information within neurons with large and spatially distinct dendritic trees (see also AGR in lobster; Combes et al., 1993), it also indicates that simply by invading areas where sensory stimuli are encoded, APs can alter sensory responses. The implications of antidromic ectopic APs for stimulus encoding should be distinct from backpropagating APs originating from the axon initial segment though. Since APs initiated at the axon initial segment are always elicited *after* dendritic stimulus integration, backpropagation can only affect future stimulus encoding. In contrast, our results demonstrate that a direct modulation of remotely initiated, backpropagating APs by modulatory neurons can affect the information encoding capability of sensory dendrites. The modulation of ectopic APs is independent of previous dendritic events, and can thus modulate incoming sensory information without prior dendritic activation.

## Synthesis

Reviewing Editor: Elizabeth Cropper, Mt Sinai Sch Med Decisions are customarily a result of the Reviewing Editor and the peer reviewers coming together and discussing their recommendations until a consensus is reached. When revisions are invited, a fact-based synthesis statement explaining their decision and outlining what is needed to prepare a revision will be listed below. Note: If this manuscript was transferred from JNeurosci and a decision was made to accept the manuscript without peer review, a brief statement to this effect will instead be what is listed below.

The authors have addressed all relevant comments from the previous review.
